# SLC6A14 Impacts Cystic Fibrosis Lung Disease Severity *via* mTOR and Epithelial Repair Modulation

**DOI:** 10.3389/fmolb.2022.850261

**Published:** 2022-03-09

**Authors:** Julia Mercier, Claire Calmel, Julie Mésinèle, Erika Sutanto, Fatiha Merabtene, Elisabeth Longchampt, Edouard Sage, Anthony Kicic, Pierre-Yves Boëlle, Harriet Corvol, Manon Ruffin, Loïc Guillot

**Affiliations:** ^1^ Sorbonne Université, Inserm, Centre de Recherche Saint Antoine, CRSA, Paris, France; ^2^ Sorbonne Université, Inserm, Institut Pierre Louis D'épidémiologie et de Santé Publique, IPLESP, APHP, Hôpital Saint-Antoine, Paris, France; ^3^ Telethon Kids Institute, University of Western Australia, Nedlands, WA, Australia; ^4^ School of Population Health, Curtin University, Bentley, WA, Australia; ^5^ Service D'Anatomie Pathologique, Hôpital Foch, Suresnes, France; ^6^ Départment de Chirurgie Thoracique et Transplantation Pulmonaire, Hôpital Foch, Suresnes, France; ^7^ UMR 0892 UVSQ-INRAE, VIM, Université Paris-Saclay, Jouy-en-Josas, France; ^8^ Centre for Cell Therapy and Regenerative Medicine, Medical School, The University of Western Australia, Nedlands, WA, Australia; ^9^ Department of Respiratory and Sleep Medicine, Perth Children’s Hospital, Nedlands, WA, Australia; ^10^ AP-HP, Hôpital Trousseau, Service de Pneumologie Pédiatrique, Paris, France

**Keywords:** cystic fibrosis, lung function, modifier genes, SLC6A14, amino acid transporter, bronchial epithelial cells

## Abstract

Cystic fibrosis (CF), due to pathogenic variants in *CFTR* gene, is associated with chronic infection/inflammation responsible for airway epithelium alteration and lung function decline. Modifier genes induce phenotype variability between people with CF (pwCF) carrying the same *CFTR* variants. Among these, the gene encoding for the amino acid transporter SLC6A14 has been associated with lung disease severity and age of primary airway infection by the bacteria *Pseudomonas aeruginosa*. In this study, we investigated whether the single nucleotide polymorphism (SNP) rs3788766, located within *SLC6A14* promoter, is associated with lung disease severity in a large French cohort of pwCF. We also studied the consequences of this SNP on *SLC6A14* promoter activity using a luciferase reporter and the role of SLC6A14 in the mechanistic target of rapamycin kinase (mTOR) signaling pathway and airway epithelial repair. We confirm that *SLC6A14* rs3788766 SNP is associated with lung disease severity in pwCF (*p* = 0.020; *n* = 3,257, pancreatic insufficient, aged 6–40 years old), with the minor allele G being deleterious. In bronchial epithelial cell lines deficient for *CFTR*, *SLC6A14* promoter activity is reduced in the presence of the rs3788766 G allele. SLC6A14 inhibition with a specific pharmacological blocker reduced ^3^H-arginine transport, mTOR phosphorylation, and bronchial epithelial repair rates in wound healing assays. To conclude, our study highlights that *SLC6A14* genotype might affect lung disease severity of people with cystic fibrosis *via* mTOR and epithelial repair mechanism modulation in the lung.

## Introduction

Cystic fibrosis (CF), the most common lethal autosomal recessive genetic disease in the Caucasian population, results from pathogenic variants in the CF transmembrane conductance regulator (*CFTR*) gene (Kerem et al., 1989; Riordan et al., 1989; Rommens et al., 1989). Manifestations of CF occur in several organs including the pancreas, the liver, and the intestine, but lung disease is the main cause of morbidity and mortality in people with CF (pwCF). CF lung disease is characterized by chronic airway colonization with microorganisms, including the most common CF life-threatening pathogen *Pseudomonas aeruginosa*, exacerbated inflammation, and lung tissue damage due to abnormal repair of the airway epithelium (Shteinberg et al., 2021).

Variability in the clinical phenotype of pwCF carrying identical *CFTR* variants and living in the same environment involves genetic modifiers, which are expected to contribute to almost 50% of CF lung phenotype (Cutting, 2015). In a large cohort of pwCF (*n* = 6,365), we previously identified five CF lung disease modifier loci by genome-wide association studies (GWAS) (Corvol et al., 2015), including one containing the solute carrier family six member 14 gene (*SLC6A14*, also known as *ATB* ^*0,+*^). *SLC6A14* is located on chromosome X and encodes for the neutral and cationic amino acid transporter SLC6A14 that concentrates amino acids into cells (with the exception of proline, glutamate, and aspartate) by using a sodium and chloride electrochemical gradient (Sloan and Mager, 1999; Jain-Vakkalagadda et al., 2004; Palazzolo et al., 2019). This transporter has been shown to be upregulated in several cancers (Ruffin et al., 2020) and to be involved in cell growth, proliferation, and the mechanistic target of rapamycin kinase (mTOR) pathway in breast (Karunakaran et al., 2011), pancreatic (Coothankandaswamy et al., 2016), and colonic (Sikder et al., 2020) cancer cell lines.

The *SLC6A14* gene has pleiotropic effects in pwCF (Ruffin et al., 2020), with several *SLC6A14* single nucleotide polymorphisms (SNPs) being associated with different phenotypes such as meconium ileus (MI) occurrence, a severe neonatal intestinal obstruction (Sun et al., 2012), lung disease severity (Li et al., 2014; Corvol et al., 2015; Pereira et al., 2017), and age at first *P. aeruginosa* infection (Li et al., 2014; Pereira et al., 2017). In particular, the rs3788766 SNP, located within the promoter region of *SLC6A14*, has been previously associated with both MI and lung function variability in pwCF (Sun et al., 2012; Li et al., 2014; Pereira et al., 2017). However, the functional consequences of this SNP on SLC6A14 expression and function are still unknown.

Here, we first analyzed the association between *SLC6A14* rs3788766 SNP and lung function by genotyping a large French cohort of pwCF. Then, we studied how rs3788766 regulate transcriptional activity of the *SLC6A14* promoter. Finally, we investigated the possible consequences of SLC6A14 activity modulation in CF bronchial epithelial cells.

## Materials and Methods

### Genotype-Phenotype Association Study

#### Participants

As of 31 January 2021, 4,975 pwCF had been included in the French CF modifier gene study (Corvol et al., 2015). The study was approved by the French Human Ethics Committee (CPP n°2004/15), and information was collected by the *Commission Nationale de L’informatique et des Libertés* (n°04.404). Informed written consent was obtained from each participant and/or parents or guardians. For the analysis, we excluded pancreatic-sufficient participants since they had milder disease (*n* = 820), non-genotyped participants for the *SLC6A14* rs3788766 SNP (*n* = 436), and participants without forced expiratory volume in 1 s (FEV_1_) measurements (*n* = 177). We also excluded participants under 6 years of age, since their spirometry data was less reliable, and those over 40 years of age, in order to limit selective survival bias (*n* = 285).

#### Lung Function and Genotyping

Measurements of FEV_1_ were collected on a quarterly basis according to international CF care recommendations (Castellani et al., 2018) and expressed as percent-predicted values (ppFEV_1_) using Global Lung Function Initiative (GLI) equations (Quanjer et al., 2012). To assess the lung disease severity, FEV_1_ were transformed to the Survival Adjusted Kulich Normalized (SaKnorm Z-value) CF-specific lung phenotype. SaKnorm is a quantitative phenotype that allows direct comparison of lung phenotypes between pwCF and accounts for differential survival (Kulich et al., 2005). Lung function and lung disease severity were analyzed over the last 3 years, except for post–lung transplant patients and patients under CFTR modulator therapy (ivacaftor and lumacaftor-ivacaftor) for whom FEV_1_ measurements were analyzed over the 3 years prior to the event. *SLC6A14* rs3788766 SNP was genotyped using Kompetitive Allele Specific PCR (KASP) chemistry (LGC, Teddington, United Kingdom).

### Immunohistochemistry

Human lung biopsy was obtained from the lung explant of a 29-year-old male with CF (homozygous for the F508del variant), after lung transplantation that occurred in the Hôpital Foch, Suresnes 92150, France. Biopsy was collected and processed in compliance with the current French public health legislation (articles L.1235-2 and L.1245-2, code de la santé publique, www.legifrance.gouv.fr). The institution informed the participant and made sure that he was not opposed to the use of surgical samples for research purposes. Staining was performed using 5-μm-thick paraffin sections from formalin-fixed paraffin-embedded lung biopsies. Immunolabeling for SLC6A14 was performed on a Bond-III^®^ automat (Leica, Leica Biosystems, Nussloch, Germany) using anti-SLC6A14 antibody (PA5-51855, Invitrogen, Carlsbad, CA, United States; 1/100).

### Reagents

SLC6A14 inhibitor α-methyltryptophan (α-MT; M8377 was from Sigma-Aldrich, Saint-Quentin Fallavier, France) was solubilized in 100% methanol (MeOH) and then diluted in the respective culture media to achieve 1, 2.5, or 5 mM (used concentrations are specified in figure legends). Equivalent volumes of MeOH alone were used for control conditions, thus reaching 0.87, 2.18, and 4.36%, respectively.

### Cell Cultures

Human bronchial epithelial cell lines Calu-3-*CFTR*-WT and Calu-3 *CFTR*-KD, kindly provided by Dr. Marc Chanson (Bellec et al., 2015) (University of Geneva, Geneva, CH), were cultured in 75-cm^2^ flasks (TPP, Techno Plastic Products, Trasadingen, Switzerland) in MEM-Glutamax (Invitrogen) medium supplemented with SVF 10% (Eurobio, Courtaboeuf, France), penicillin-streptomycin (100 U/mL, Invitrogen), sodium pyruvate 5% (Invitrogen), essential amino acids 1 mM (Invitrogen), and HEPES buffer 10 mM (Thermo Scientific, Waltham, Massachusetts, United States). Calu-3 cells were then seeded in plates with 12 (3–3.5×10^5^ cells/well) or 24 (2×10^5^ cells/well) wells (TPP) and maintained at 37°C in a humidified atmosphere with 5% CO_2_. Non-CF and CF primary human bronchial epithelial cells (HBECs) (Epithelix, Plan-les-Ouates, Switzerland) (characteristics in Table 1) were grown on 12- or 24-well plates (TPP) (1 × 10^5^ cells/well) until confluent in hAEC culture medium supplemented with antibiotics (Epithelix).

**TABLE 1:** Characteristics of the donors from whom the primary bronchial epithelial cells were obtained.

Patient	Age	Sex	Smoker	Pathology	*CFTR* variant	Figure
CFAB060901	21	Female	No	CF	F508del/F508del	Figures 3A,B,C
CFAB056701	39	Female	No	CF	F508del/F508del	Figure 3C
CFAB45202	32	Male	No	CF	F508del/F508del	Figure 3B
CFAB064901	37	Female	No	CF	F508del/1717-1G>A	Figure 3C
02AB77201F2	63	Male	No	None	-	Supplementary Figure S1B
02AB067101	72	Male	No	None	-	Supplementary Figures S3A, S3B
02AB068001F2	71	Female	No	None	-	Supplementary Figures S3A, S3B
02AB083901	59	Male	No	None	-	Supplementary Figure S3B

### *SLC6A14* Promoter Activity

Gaussia luciferase (GLuc) reporters driven by the *SLC6A14* promoter harboring either the A or G allele of rs3788766 (GeneCopoeia, Rockville, MD, United States) were used (sequences available in Supplementary Material). Calu-3-*CFTR*-KD cells were seeded in 24-well plates and transfected at 60% confluence with 1 μg/ml of the GLuc *SLC6A14* promoter reporter using Lipofectamine 3,000 (Invitrogen). After 24 h of transfection, culture media were collected and centrifuged for 10 min at 10,000x *g*, and supernatants were stored at –20°C. *SLC6A14* promoter activity was quantified by measuring Gaussia luciferase and secreted embryonic alkaline phosphatase (SEAP) used as endogenous reporter and was measured using a Secrete-Pair™ Dual Luminescence Assay kit (GeneCopoeia). *SLC6A14* promoter activity is represented as the ratio of GLuc normalized by SEAP.

### L-Arginine Uptake Quantification

SLC6A14 amino acid transport was studied as described by others (Di Paola et al., 2017). Briefly, cells cultured in 12-well plates were washed and then incubated in HEPES buffer (25 mM HEPES, 140 mM NaCl, 5. 4mM KCl, 1.8 mM CaCl_2_, 0.8 mM MgSO_4_, 5 mM glucose, pH = 7.4) for 30 min at 37°C. Cells were then incubated with 300 µl of HEPES buffer supplemented with 100 µM Arginine and Arginine Monohydrochloride L-[2,3,4-^3^H] (L-Arginine-2,3,4-^3^H, 1 μCi/ml, specific activity: 54.5 Ci/mmol, lot: 2422780, PerkinElmer, Villebon-sur-Yvette, France) for 15 min and washed 3 times on ice with ice-cold HEPES with 10 mM Arginine (inhibition of uptake). Finally, cells were lysed with 400 µl 0.5M NaOH for 15 min while shaking on ice. Radioactivity levels were measured in 300 µl of the sample in 7 ml of a scintillation liquid, Ecolite Plus (MP Biomedicals, Illkirch-Graffenstaden, France), and using Hidex 300SL (LabLogic ScienceTec, Villebon-sur-Yvette, France) equipment.

### Wound-Healing Assay

Cell monolayers grown on plastic supports were injured mechanically (3 wounds per well) as previously described (Ruffin et al., 2016; Valera et al., 2019). Afterward, cells were washed with their culture medium to remove detached cells and treated with either α-MT (1, 2.5 or 5 mM) or MeOH which acted as the vehicle control (respective equivalent volumes of MeOH alone). Photographs of the wounds were taken at two different positions on each wound using an inverted microscope with an X4 objective at t = 0 h and t = 6 h post-wounding. Images were analyzed using ImageJ software (https://imagej.nih.gov/ij/index.html) to measure areas of the wounds at t = 0 h and t = 6 h (end-point assay), and mean wound closure (% of the area at t = 0 h) was calculated (wound closure = T0-T6/T0). Statistical analysis is performed on raw data. Data illustrated are reported to one MeOH (1 mM equivalent) control.

### Cytotoxicity Measurement

Toxicity of α-MT was verified using a commercially available assay (CytoTox 96^®^ Non-Radioactive Cytotoxicity Assay, Promega, Madison, WI, United States).

### Protein Extraction and Western Blot

Protein extracts (20 µg) in RIPA buffer supplemented with antiprotease-antiphosphatase (Halt™ Protease and Phosphatase Inhibitor Single-Use Cocktail, Thermo Scientific) were reduced and size-separated on 4-15% Mini-PROTEAN^®^ TGX Stain-Free™ Precast Gels (Bio-Rad, Hercules, CA, United States) and transferred onto nitrocellulose membranes using an iBlot2™ Gel Transfer Device and iBlot2™ Nitrocellulose Regular Stacks (IB23001, Invitrogen). Membranes were incubated with specific primary antibodies followed by corresponding secondary-HRP antibodies. Mouse anti–β-actin antibody (A2228) (1/1,000) was from Sigma-Aldrich. Rabbit anti–phopho-mTOR (#2971, 1/1,000), rabbit anti-mTOR (#2983, 1/1,000), anti-mouse HRP (#7076, 1/10000), and anti-rabbit (#7074, (1/5,000) antibodies were from Cell Signaling Technology (Denver, CO, United States). Immunodetection was carried out using Clarity™ Western ECL Substrate (#170-5,061, Bio-Rad). Image acquisition was performed using Las-3000 (Fujifilm, Bussy-Saint-Georges, France). Densitometric quantification was performed using ImageJ software.

### Statistical Analysis

#### Genotype-Phenotype Analysis

Descriptive statistics are reported as mean ± standard deviation (SD) or percentages. Association between lung disease severity and *SLC6A14* rs3788766 genotypes was evaluated by linear regression. We applied additive SNP coding, and the reference allele (i.e., the allele with the highest frequency in the European population) was taken from annotations of the human genome (http://www.ensembl.org). Fisher’s exact test was used to test conformance of the allele frequencies with the Hardy-Weinberg equilibrium. A *p*-value of less than 5% was interpreted as evidence of a statistically significant association. Analyses were carried out using R software (version 3.6.3, http://www.R-project.org/).

#### *In vitro* Data

All data are presented as mean ± SD and the number of repeated experiments is indicated in the figure legends. GraphPad Prism version 7.05 (GraphPad Software, San Diego, CA, United States) was used to analyze all data. Paired or unpaired *t*-tests were used to compare two groups. One-way ANOVA was used for comparison of more than two groups and was followed by appropriate *post hoc* tests as indicated in the figure’s legends. Values of *p* < 0.05 were considered to be significant. In figures, statistical differences are indicated as *p* < 0.05 (*), *p* < 0.01 (**), and *p* < 0.001 (***) or non-significant (NS).

## Results

### *SLC6A14* rs3788766 SNP Is Associated With Lung Function in People With CF

Among the 4,975 pwCF included in the French CF modifier gene study (i.e., 70% of French pwCF) and after application of the exclusion criteria, 3,257 pwCF were analyzed in the genotype/phenotype association study. Demographic characteristics of the participants are summarized in Table 2. The minor allele frequency (MAF) of *SLC6A14* rs3788766 SNP in our cohort was 0.38, similar to that reported in Europeans (0.36). As *SLC6A14* is located on the X chromosome, the Hardy-Weinberg equilibrium *p*-value was computed by Fisher’s exact test among females. Results showed that our cohort does not significantly diverge from the Hardy-Weinberg equilibrium (*p*-value = 0.459). We also found that *SLC6A14* rs3788766 SNP was associated with lung function with the G allele being deleterious. Linear regression models estimated that pwCF carriers of the minor allele G had a significant increase in lung disease severity, which was measured by an average loss in the SaKnorm Z-value of 0.038 ± 0.016 for each G allele (*p* = 0.020) (Table 3). Overall, an average decrease of ∼1.5% of ppFEV_1_ was observed in patients carrying at least one rs3788766 G allele (Table 3).

**TABLE 2:** Demographic and clinical characteristics of 3,257 patients with cystic fibrosis analyzed in the phenotype-genotype study.

Characteristics	Patients analyzed in SLC6A14 rs3788766 study
Age at inclusion (years), mean ± SD	21.5 ± 8.4
Females, % (n)	49% (1,581)
Caucasian origin, % (n)	91% (2,976)
Lung transplant, % (n)	17% (567)
CFTR modulator therapy^a^, % (n)	29% (935)
CFTR genotypes, % (n)	
F508del homozygous	54% (1750)
F508del heterozygous	35% (1,154)
Others	11% (353)

a Patients who have started CFTR, modulator therapy with ivacaftor or lumacaftor-ivacaftor. *CFTR*: cystic fibrosis transmembrane conductance regulator.

**TABLE 3:** Genotype-phenotype association study between lung disease severity and *SLC6A14* rs3788766 genotypes, in 3,257 patients with cystic fibrosis.

*SLC6A14* rs3788766 genotype*s*	Patients analyzed% (n)	Lung function ppFEV_1_ ^a^ mean ± SD	Lung disease severity SaKnorm Z-value^b^ mean ± SD	*p*-value^c^
AA	50% (1,624)	64.9 ± 27.2	0.360 ± 0.795	0.020
AG	23% (740)	63.5 ± 25.7	0.302 ± 0.797	
GG	27% (893)	63.1 ± 26.4	0.287 ± 0.780	

a PpFEV_1_: Percent-predicted (pp) Forced expiratory volume in one second (FEV_1_).

b SaKnorm: Survival adjusted Kulich Normalized.

c *p*-value was computed by linear regression with additive model.

### *SLC6A14* rs3788766 Genotype Affects *SLC6A14* Promoter Activity

**FIGURE 1:**
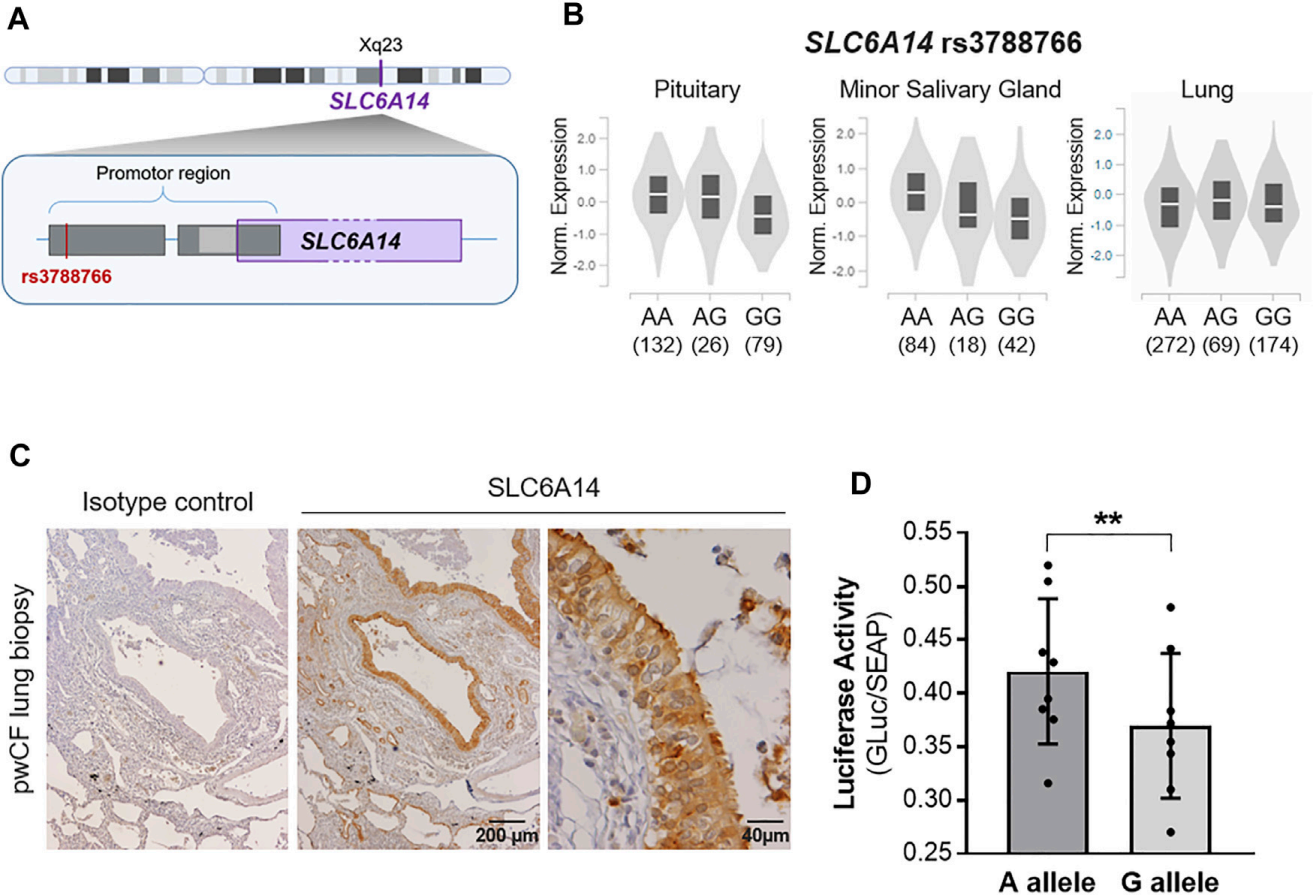
*SLC6A14* rs3788766 variant is associated with gene transcription regulation. **(A)** Graphical representation of the localization of rs3788766 and *SLC6A14* gene on X chromosome. **(B)** Violin plots of *SLC6A14* transcript expression in tissues according to rs3788766 genotypes in Genotype-Tissue Expression (GTEx, https://www.gtexportal.org/home/). **(C)** Representative images of SLC6A14 immunohistochemistry on pwCF lung biopsy. **(D)** *SLC6A14* promoter activity measurement was performed on Calu-3-*CFTR*-KD transfected with the reporter plasmid constructs containing A allele or G allele of rs3788766 (*n* = 8 independent experiments) (Wilcoxon test, **p < 0.01).

The *SLC6A14* rs3788766 SNP, located within *SLC6A14* promoter (Figure 1A), is likely to affect *SLC6A14* mRNA expression. Quantitaive-trait Loci (QTL) expression data extracted from GTEx (V8 release, https://www.gtexportal.org/home/) show that rs3788766 G is associated with a decrease in *SLC6A14* transcript expression in different tissues (Figure 1B), including the pituitary (*p*-value = 3.0 × 10^−8^) and the minor salivary gland (*p*-value = 1.8 × 10^-5^). In the lung, a diminished but not statistically significant expression is observed (AA vs. GG genotypes). It is worth mentioning that for the GTEx project, the prefered location for the lung tissue collection is in the inferior segment of the left upper lobe, 1 cm below the pleural surface, avoiding any large arteries, veins, and bronchi. We observed by immunohistochemistry that SLC6A14 is predominantly expressed in the bronchial epithelium of pwCF (Figure 1C). Therefore, *SLC6A14* expression using QTL analysis might not inform about SNP consequences on *SLC6A14* expression in the lung. Thus, to determine whether rs3788766 affects *SLC6A14* promoter activity in bronchial epithelial cells, we used *SLC6A14* promoter reporters carrying either the A or the G allele of this SNP and assessed the reporter expression. We observed that Calu-3-*CFTR*-KD cells transfected with *SLC6A14* promoter reporter plasmid carrying the G allele had a lower luciferase activity (12.1% reduction) than cells transfected with the A allele (Figure 1D). This result indicates that the G allele of rs3788766, that is, the minor allele, is associated with a decreased *SLC6A14* promoter activity.

### SLC6A14 Inhibition in Human Bronchial Epithelial Cells Regulate mTOR Phosphorylation and Epithelial Repair

**FIGURE 2:**
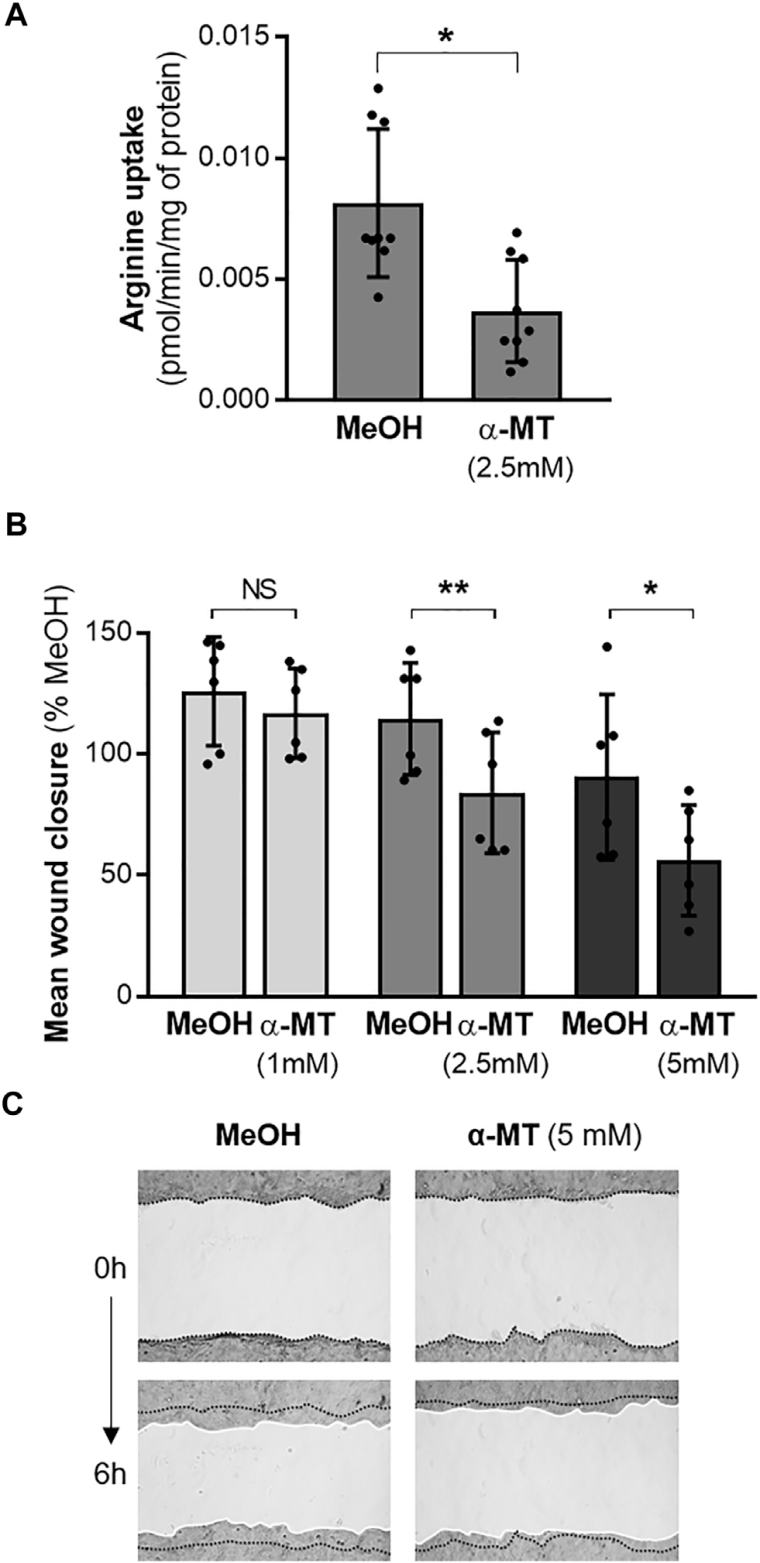
Effect of SLC6A14 inhibition in Calu-3-*CFTR*-KD cells. **(A)** ^3^H-Arginine uptake in Calu-3-*CFTR*-KD cells treated with α-MT (2.5 mM) or vehicle (MeOH) (n = 9 independent experiments, Wilcoxon test, *p < 0.05). **(B)** Calu-3-*CFTR*-KD were treated with increasing doses of α-MT or vehicle (MeOH) for 6 h. Quantification of wound closure expressed in mean % compared to the control condition (*n* = 6 independent experiments, ANOVA followed by Sidak’s multiple comparisons test, *p < 0.05, **p < 0.01). **(C)** Representative images of wounds at 0 and 6 h in control (MeOH) and α-MT conditions. Wounds have been brightened on the pictures and white lines have been drawn at the wound edges for a better visualization.

**FIGURE 3:**
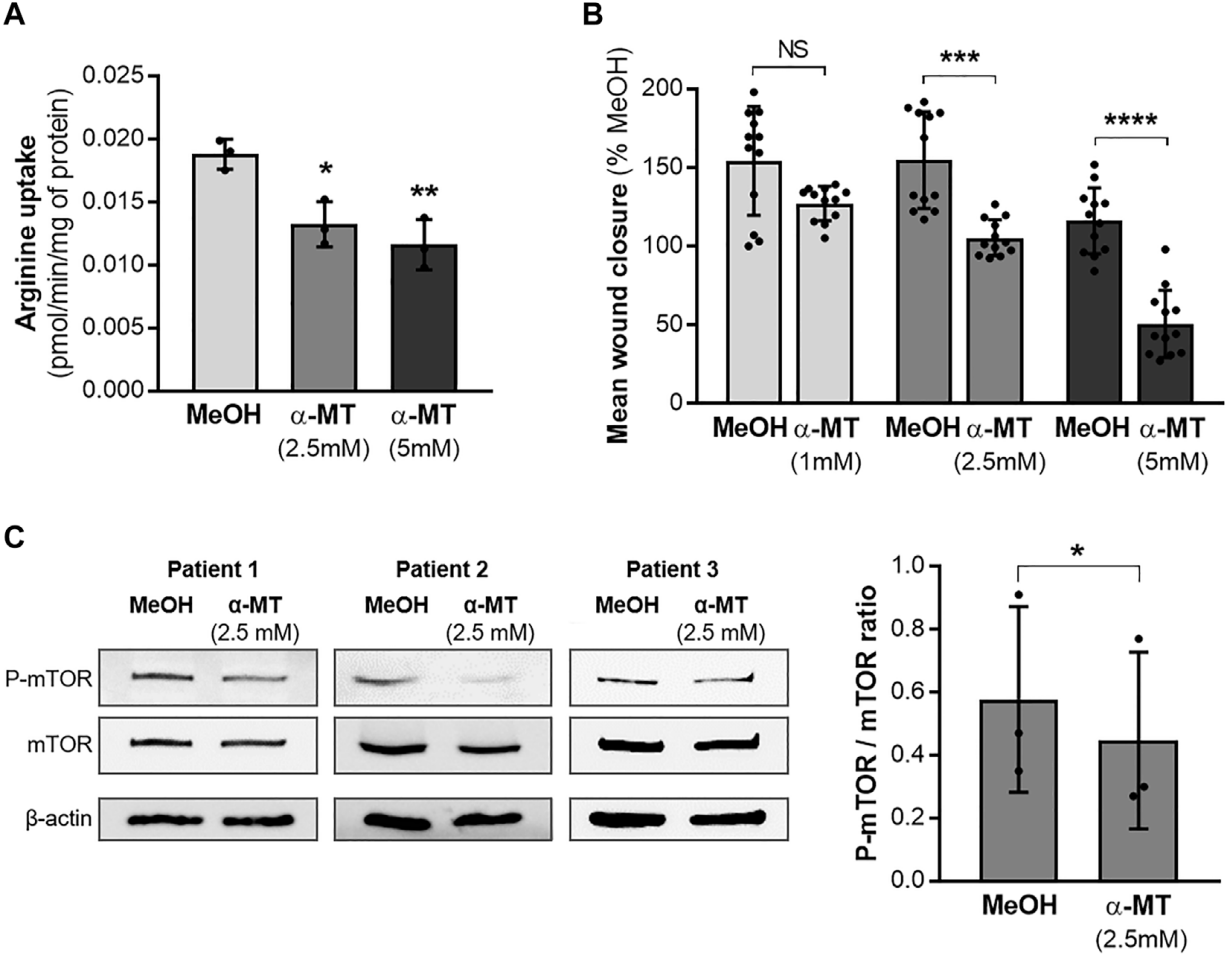
Effect of SLC6A14 inhibition in CF primary human bronchial epithelial cells (HBECs). **(A)** ^3^H-arginine uptake in CF primary HBEC treated or not with α-MT (n = 3 independent experiments realized with the cells from one CF donor), ANOVA followed by Dunnett’s multiple comparisons test, *p < 0.05, **p < 0.01). **(B)** Measurement of epithelial repair (6 h) of CF primary HBECs treated with increasing doses of α-MT or vehicle (MeOH). Quantification of wound closure is expressed in mean % compared to the control condition (*n* = 12 independent experiments realized with the cells from two CF donors (6 per donor)), ANOVA followed by Sidak’s multiple comparisons test, ***p < 0.001, ****p < 0.0001). **(C)** Western blot, images of phospho-mTOR, total mTOR, and β-actin (loading control) (left) and quantification of P-mTOR/mTOR ratio (right) in CF primary HBEC from three different CF donors treated for 6 h with 2.5 mM α-MT or control vehicle (MeOH). Paired *t*-test *p < 0.05.

Since the *SLC6A14* rs3788766 G allele is likely to reduce *SLC6A14* mRNA expression level and consequently its activity as an amino acid transporter, we sought to investigate the cellular consequences of a decreased activity of SLC6A14 in CF bronchial epithelial cells. Thus, we inhibited its activity in bronchial epithelial cells using α-MT, a specific pharmacological blocker of SLC6A14 (Karunakaran et al., 2008). First, we measured LDH release to ensure that α-MT was not toxic at the doses used (Supplementary Figure S1). We determined that cytotoxicity levels in Calu-3-*CFTR*-KD treated with 1, 2.5, and 5 mM of α-MT were similar to those of cells treated with the vehicle (Supplementary Figure S1A). Then, we showed that α-MT induced a 54% decrease of ^3^H-Arginine transport in Calu-3-*CFTR*-KD cells (Figure 2A). A similar effect was observed in Calu-3-*CFTR*-WT (Supplementary Figure S2A). As SLC6A14 is involved in colon cancer cell proliferation, migration, and invasion (Sikder et al., 2020), we wondered whether it could play a role in bronchial epithelial repair, a process which involves both cell proliferation and migration mechanisms. Thus, we performed scratch assay experiments on Calu-3-*CFTR*-KD monolayers, treated or without α-MT at t = 0 h and for the following 6 h of repair (Figures 2B,C). Quantitative analysis highlighted a dose-dependent inhibition of wound closure with decreases of 8, 27, and 39%, at 1, 2.5, and 5 mM of α-MT, respectively (Figure 2B). In contrast, no significant wound closure inhibition was observed at 1 and 2.5 mM of α-MT in Calu-3-*CFTR*-WT, but a 31% decrease in wound closure was observed at 5 mM of α-MT (Supplementary Figure S2B). To ensure that this effect was not restricted to the Calu-3 cell lines, we performed similar experiments in primary HBECs isolated from patients with CF homozygous for the F508del *CFTR* variant (Figure 3B) or from healthy subjects (Supplementary Figure S3A). Significant decreases of 25 and 36% of arginine transport were observed in CF HBECs treated with 2.5 and 5 mM of α-MT, respectively (Figure 3A). Similar to the Calu-3 cells, no increase in cytotoxicity has been observed in non-CF HBECs, treated with α-MT or not (Supplementary Figure S1B). A decrease of 29 and 55% of wound closure after 6 h of repair was observed in CF HBECs with 2.5 and 5 mM of α-MT (Figure 3B), respectively, while a 73% decrease was found in non-CF HBECs with 5 mM of α-MT (Supplementary Figure S3A). Finally, we wondered if SLC6A14 amino acid transport inhibition could have an impact on mTOR activity in primary bronchial epithelial cells, as it was previously shown in pancreatic and colonic cells (Coothankandaswamy et al., 2016; Sikder et al., 2020). Therefore, we evaluated mTOR activation by Western blot in primary HBECs treated or not treated with 2.5 mM of α-MT. From results generated, we observed that SLC6A14 activity inhibition induces a significant decrease in mTOR phosphorylation in primary CF (Figure 3C) cells. A similar effect is observed in non-CF (Supplementary Figure S3B) HBECs, however, without reaching significance.

## Discussion

Lung disease severity is highly variable among pwCF, with *CFTR*, the environment, and modifier genes all contributing to this variability. Among the modifier genes, *SLC6A14* is of particular interest because it has been associated with both lung and digestive phenotypes in pwCF (Sun et al., 2012; Li et al., 2014; Corvol et al., 2015; Pereira et al., 2017). This suggests a major pleiotropic role of SLC6A14 in the overall pathophysiology of the disease (Ruffin et al., 2020). Here, we confirmed the association between *SLC6A14* rs3788766 SNP and the lung function of pwCF and further demonstrated that carrying the minor allele of rs3788766 induces a decreased *SLC6A14* promoter activity. We finally demonstrated that a reduced SLC6A14 amino acid transport activity alters wound repair mechanisms and modulates the mTOR pathway in human CF bronchial epithelial cells.

Our study first showed, using a large French CF cohort (*n* = 3,257), that pwCF carrying at least one minor allele G of the *SLC6A14* rs3788766 SNP exhibit reduced lung function compared to those carrying two major allele A, confirming prior studies (Li et al., 2014; Pereira et al., 2017). Li *et al.* performed a sub-analysis from the original 1,661 Canadian CF Gene Modifier Study participants of a previous study (Sun et al., 2012) and showed that rs3788766 is associated with both pediatric lung disease severity and earlier age at first acquisition of *P. aeruginosa* (Li et al., 2014). Other *SLC6A14* SNPs, also associated with the lung function of pwCF, have been identified either by GWAS (Corvol et al., 2015) or genotyping (Ruffin et al., 2020). Beside lung phenotype, Sun *et al.* showed that the rs3788766 SNP is associated with digestive manifestations of CF, such as increased MI susceptibility in a cohort of 3,763 pwCF (Sun et al., 2012). Those results were further confirmed in a genome-wide association investigation performed by the International CF Gene Modifier Consortium with 6,770 pwCF (Gong et al., 2019). Altogether, these results emphasize the major involvement of this particular SNP of *SLC6A14* in CF clinical variability.

To understand how this SNP can contribute to CF pathophysiology and because of its location within the *SLC6A14* regulatory region, we evaluated its impact on *SLC6A14* promoter activity. We found that the minor allele G, previously identified as the deleterious allele regarding the CF patient’s lung function, is associated with a decrease in *SLC6A14* promoter activity in bronchial epithelial cells. This is in contradiction to eQTL data in GTEX lung samples. However, as evocated above, this discrepancy likely results from GTEX lung sample collection avoiding bronchi. In addition, SLC6A14 eQTLs were already shown not to colocalize with lung GWAS associated evidence (Gong et al., 2019). This is the first report showing that a *SLC6A14* SNP might influence *SLC6A14* transcription in the context of CF. Indeed, SLC6A14 expression and function have been mostly investigated in cancers so far (Sikder et al., 2017). It is worth mentioning that using a similar method, a recent report has shown that the obesity-associated *SLC6A14* rs2011162 SNP also reduced *SLC6A14* expression (Sivaprakasam et al., 2021).

Recently, some studies have explored the role of SLC6A14 in CF pathophysiology and begun to explain the reasons for its identification as a modifier gene of CF lung and intestinal diseases. Di Paola *et al.* showed that the inhibition of SLC6A14 amino acid transport increased *P. aeruginosa* attachment to human bronchial epithelial cells by enhancing L-arginine levels in the airway surface liquid (Di Paola et al., 2017). Arginine transport through SLC6A14 also seems to increase F508del-CFTR protein by enhancing nitric oxide (NO) production and activating cGMP or PKG pathways (Ahmadi et al., 2019b). NO production increase has also been suggested to contribute to anti-infectious response because it is well known to have bactericidal effects on *P. aeruginosa* (Hibbard and Reynolds, 2019). Thus, SLC6A14 seems to modulate CFTR activity and could participate in the infectious process of CF airways by *P. aeruginosa*. Concerning the role of SLC6A14 in the intestine, it has been suggested that SLC6A14 involvement in MI susceptibility could be related to intestinal fluid secretion defect in CF, which was worsened in *Slc6a14*-KO CF mice carrying the major mutation F508del (Ahmadi et al., 2018).

Here, we report, for the first time, that SLC6A14 is involved in bronchial epithelial repair. In healthy epithelia, repair processes involving cell proliferation, migration, and differentiation facilitate epithelial integrity restoration and function. In CF, repair mechanisms are altered and chronic infections with various pathogens and exacerbated inflammation induce progressive epithelial damage (Ruffin and Brochiero, 2019). Previous studies have also shown that wound healing is delayed in CF epithelia compared with non-CF controls (Schiller et al., 2010; Trinh et al., 2012), highlighting an important role of CFTR in cell differentiation and regeneration (Amaral et al., 2020). However, the specific involved cellular signaling pathways related to CFTR remain to be determined. Our results demonstrated that in Calu-3 cells and primary HBEC, pharmacological inhibition of SLC6A14 activity resulted in a delayed epithelial repair. It was recently shown that SLC6A14 expression and function are similar in non CF and CF primary bronchial cells, and arginine uptake *via* SLC6A14 increases the function of F508del-CFTR and involves the NO synthase pathway (Ahmadi et al., 2019a). Whether the observed delayed epithelial repair involves this NO synthase pathway remains to be determined. SLC6A14 involvement in cell migration and proliferation has been previously described. Indeed, Sikder *et al.* first showed that SLC6A14 function favors cell proliferation and invasion in colon cancer LS174T cell line (Sikder et al., 2020). In addition, Mao *et al.* showed that SLC6A14 overexpression or knockdown, respectively, promotes or inhibits migration and proliferation of colorectal cancer cells (HCT-116 and Caco-2 cells) *in vitro* (Mao et al., 2021). They also found that the pharmacological inhibitor α-MT inhibited cell proliferation and the fact that SLC6A14 promoted colorectal cancer cell proliferation and migration *via* the JAK2/STAT3 pathway. SLC6A14 involvement in cell proliferation has also been shown in other cancer cells such as pancreatic cancer cells (Coothankandaswamy et al., 2016).

In addition to its role in epithelial repair, we highlighted that it may be involved in the mTOR pathway. The mTOR pathway balances anabolism and catabolism in order to control key cellular processes such as cell growth or proliferation. It is very sensitive to amino acid starvation, especially leucine and arginine (Liu and Sabatini, 2020). SLC6A14 implication in the mTOR pathway was previously described in pancreatic cancer cell lines (Coothankandaswamy et al., 2016) for which α-MT–mediated SLC6A14 blockade induces the decreased phosphorylation of proteins involved in the mTOR pathway including 4E-BP1, eIF-2α, and S6kinase. This interplay between SLC6A14 and mTOR was also recently confirmed in colon cancer LS174T cell line treated with α-MT (Sikder et al., 2020). In the intestinal epithelium, mTOR is involved in wound healing and the re-establishment of barrier function following injury (Kaur and Moreau, 2019). Consistent with the literature, we confirmed the relation between SLC6A14 activity and mTOR activation. However, how airway epithelial repair, mTOR, and SLC6A14 are related remains unknown. Recently, SLC6A14 was shown to be a target for Wnt-signaling (Sikder et al., 2020), which is known to be one of the key pathways involved in lung repair and regeneration in response to injury (Raslan and Yoon, 2020). Further work is thus necessary to fully understand the consequences of SLC6A14 blockade on the molecules of the mTOR pathway specifically related to this wound repair process. This is particularly important as it has been shown that modulation of the mTOR pathway can influence the stability and function of CFTR (Reilly et al., 2017).

To conclude, we confirmed that *SLC6A14* rs3788766 genotype influences the lung disease severity of pwCF. This study also showed that *SLC6A14* might influence CF lung phenotype *via* the mTOR signaling pathway and epithelial repair process modulation.

## Data Availability

The original contributions presented in the study are included in the article/Supplementary Material, further inquiries can be directed to the corresponding authors.
