# Supplementary material for: SLC6A14 Impacts Cystic Fibrosis Lung Disease Severity via mTOR and Epithelial Repair Modulation
Source: Front Mol Biosci. 2022 Mar 9;9:850261. doi: 10.3389/fmolb.2022.850261 (PMC8965518; doi:10.3389/fmolb.2022.850261)

**SUPPLEMENTARY MATERIAL**

**Supplementary Material 1.** SLC6A14 promoter sequences with a G or A allele in Gluc-ON^TM^ promoter reporter clone (pEZX-PG04, genecopoeia).

TGACGTGCCCCAAGGTTGGCTTCTTTCATTTATATGGGACTTTGATCGTTTAGTAAATGCTATTGGATTTGCTTTTTAGCTCATCCTTTTATAAGGAGGTTTATAAGCCCTTCTTGCTCCTCTCCCTTCTATGTTTAATCTTAGCCTTTAGGTCATACCAGTAGTGTACAGTACTAATAGGCACACACTCATGCATTAGCACTCTCCATCCCCCAATTCCCCATTGATACATGCACATGTGCACACACACACACATGCACATCAGCCTTTGTTATGTTCAAGACAAAGTTAAATAAAACTTATTGATACTTTCCTTACTACCATCCTACATCTTTCATGGACTTTTCTCTACCTTACCTGCCAAGATTCCCCAGGGCATATTTCTATTGCAAATGGAAAATTCTTGCAGTCAGTGGAGAACAAAGGAGCT**[G/A]**TACATAGGGTACAGAATTTGCCTATTTGCTCATTCCTCTGTGTGCATGAATTTGTGCTTTGCTTCATAGAACCACCATCACTATCTGTTACCTGGGCAGACTGAGTTTAAATCCTTTGAGTTTCCTGATGAAAAGGCATTCCATTGGTAAACAGCATTATAATAATTATTTCCTCCCTGGTCAAGCTGGGATGTTTCCTCATAGTTTACTTTCTAGGCCTCATCTTTCTTACAGAGTGTGCTCCTTTGTTAAGGTTAGAATTTCCCATAAACCTGCTCAATAATTTGTTTGTGTTTGGCTTCTTTGAAATACTACACAAAGCAATCCCTGTAAAAGGCAAAGCTGTCCTGAAGGCTGAGAAAGGAGCCTGAGACATAGGCTCCAAGTTGCTCTTTTCAGGCAGAGCCAGCTGGGTAATCTTATCTCAGATGGCTGCTTTTCAAGGTGCCCAATTCAGGGGCTTTTCCTCTGGGAGCAGCATTTGCCCCAGGGAATCAAGTGCTTTCTAGTCAGGGGCAAAACTTTGGGAAATCTGAGGACCCAGGGTGGTATGGTCTGTTCAGGAGAATTTTGGGGAACAGAATGGCCCCCTTCTCCCTCCAGCACTTGTACAGATCAGCACTTGGCCCCAGAACAGAGACCAGACTGAGAGGCGAGGTTAGGAGGAAACAGGGGACCCAGGAAAGGCGGCTAGATTGCAAACGTACCTACACAGCTCTGAGTCAAAGGCTGTCAGTCATCTCGGCTCAGACTGCTCTGCTCTCCAGCAGCCCAGCCCTTTCCCAGGGCTGGGGCAGGAGATTGCTACATGTAGGCTTATCTGGGGAAAAACCAGAGCCTCACTTTAGTCCCTTCCGGTAATTGACACTACTGGACACCCAGGAGGGGGAGGAGAGAGCTTCTCTTCATAAATGTTCCCACCCCTGGGCAAGGTGGCTCACTCTGGCAGGTAGGAACAGGGGAGAGTGCACCTGCTACCAGTCAAGCTCAGCCAGACTGCAAGAGGAGGCGAGGCGGAGCCAGCCGAGGGAGT

**Supplementary Figure S1.** **Impact of α-MT on cell viability.** **A**. Cell viability of Calu-3-*CFTR*-KD cells treated with increasing doses of α-MT or vehicle (MeOH) (n=3 independent experiments, ANOVA followed by Sidak’s multiple comparisons test). **B.** Impact of α-MT on viability of non-CF primary human bronchial epithelial cells from 1 patient (n=3 independent experiments, ANOVA followed by Sidak’s multiple comparisons test).

**
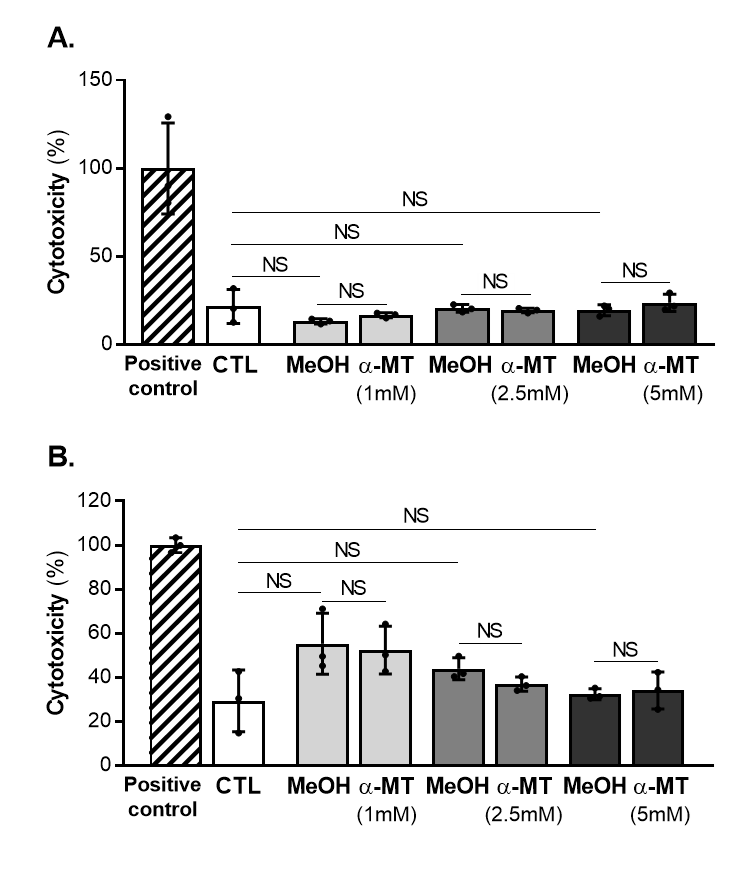
**

**Supplementary Figure S2.** **Effect of SLC6A14 inhibition in Calu-3-*CFTR*-WT cells.** **A.** ^3^H-Arginine uptake in Calu-3-CFTR-WT cells treated with α-MT (2.5 mM) or vehicle (MeOH) (n=7 independent experiments, Wilcoxon test, * p<0.05). **B.** Epithelial repair of Calu-3-*CFTR*-WT cells treated with increasing doses of α-MT or vehicle (MeOH). Quantification of wound closure is expressed in mean % compared to the control condition (n=5 independent experiments, ANOVA followed by Sidak’s multiple comparisons test, * p<0.05).

**
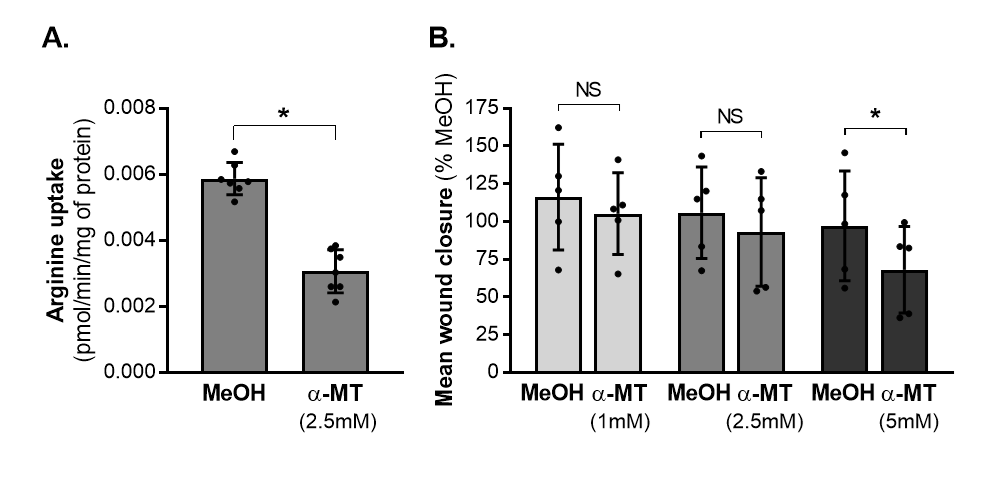
**

**Supplementary Figure S3.** **Impact of SLC6A14 inhibition in non-CF primary human bronchial epithelial cells.** **A.** Epithelial repair of non-CF primary human bronchial epithelial cells. Quantification of wound closure is expressed in mean % compared to the control condition (n=9 independent experiments realized with the cells from 2 non-CF donors (n=6 and n=3)), ANOVA followed by Sidak’s multiple comparisons test, *p<0.05). **B.** Western blot, images of phospho-mTOR, total mTOR and β-actin (loading control) (**left**) and quantification of P-mTOR / mTOR ratio (**right**) in non-CF primary HBEC, from three different non-CF donors, treated 6 h with 2.5 mM α-MT or control vehicle (MeOH), paired t test.


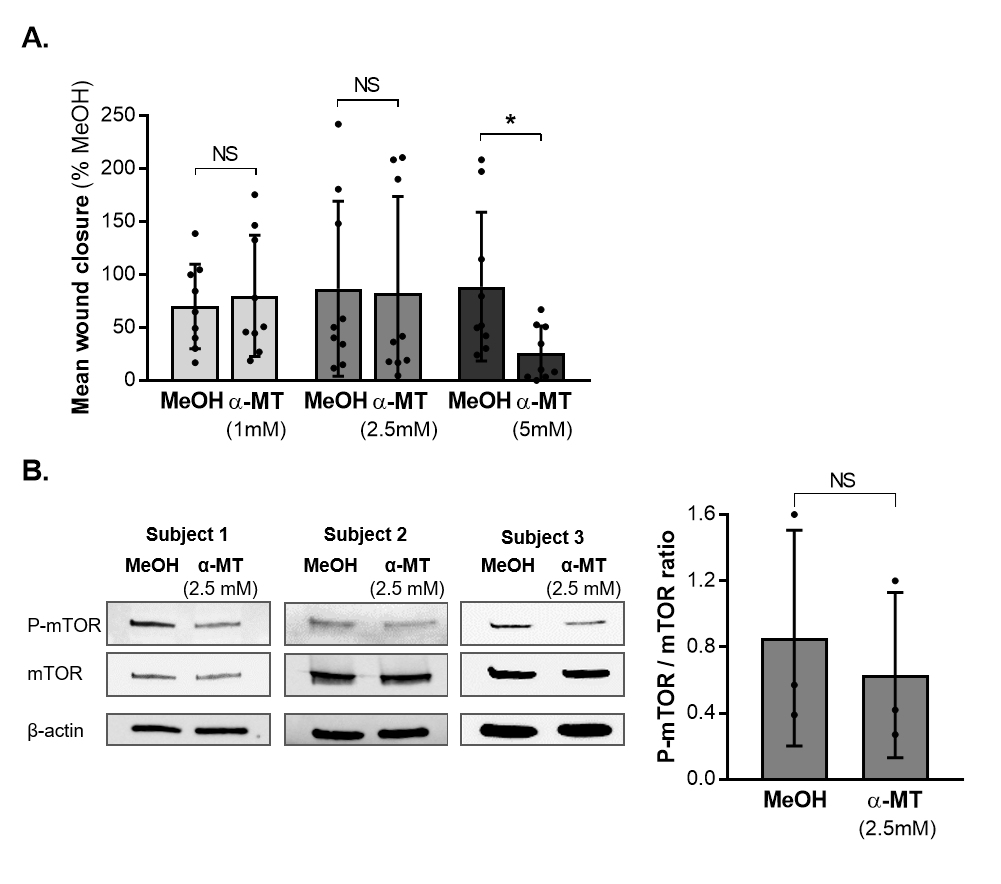

Supplement: Supplementary file 1 [file DataSheet1.DOCX]
